# Rediscovery of *Eremobittacus spinulatus* Byers (Mecoptera, Bittacidae) in Mexico, with description of the female and comments on sexual dimorphism and potential mimicry

**DOI:** 10.3897/zookeys.539.6623

**Published:** 2015-11-23

**Authors:** Fernando Villagomez, Atilano Contreras-Ramos, Yesenia Marquez-López

**Affiliations:** 1 Laboratorio de Ecología y Sistemática de Microartrópodos, Departamento de Ecología y Recursos Naturales, Facultad de Ciencias, UNAM, 04510 México, D.F., Mexico; 2 Instituto de Biología, UNAM, Departamento de Zoología, Cd. Universitaria, 04510 México, D.F., Mexico; 3 Maestría en Biología, Universidad Autónoma Metropolitana-Iztapalapa, México, D.F., Mexico

**Keywords:** Hangingfly, sexually dimorphic, tropical dry forest, taxonomy, key, mimicry

## Abstract

The female of *Eremobittacus spinulatus* Byers, 1997 is described for the first time. A key to the two species known of this genus endemic to Mexico is provided, and species distribution is illustrated. A case is made for adults of *Eremobittacus* to be sexually dimorphic, which appears to be an exceptional occurrence in Bittacidae. It is claimed that *Eremobittacus spinulatus* habitus has a wasp-like appearance, which may potentially depict a case of mimicry.

## Introduction

*Eremobittacus* was erected by Byers (1997) with *Eremobittacus spinulatus* Byers as type species. This genus is endemic to Mexico and includes a second species, *Eremobittacus sodalium* (Byers 2011); each described from a single male specimen. Immature stages and females have remained unknown, because of an apparent rarity of these species. Byers (1997) comments that he desired for additional specimens to achieve a more complete description of the genus, as well as an accurate phylogenetic placement. Byers and other entomologists visited again *Eremobittacus spinulatus* type locality and were unable to find additional specimens.

*Eremobittacus* shares several traits with the widespread *Bittacus*, as well as with *Harpobittacus*, an endemic of Australia. Alignment of longitudinal veins in three slightly pigmented columns, wing venation, body microsculpture, particularly of hind femora, and coloration, were used as diagnostic traits for the genus. However, after *Eremobittacus sodalium* was described, the genus diagnosis was slightly modified: vein A1 long, with its distal end beyond origin of M, basitarsus of hind leg almost the same length of tarsomeres II and III together, noticeable thickening of hind femur, basistyle short and bulging, dististyle with setae only on margin, cerci very short and aedeagus uncoiled (Byers 2011).

In Bittacidae, most diagnostic characters used for species differentiation are found in the male genitalia. From species descriptions (e.g., Byers 1996, 2004) it becomes evident that modifications in shape and size of abdominal sclerites of the female might also help in species identification. Nonetheless, we have little female genitalia diagnostic information available for other bittacid species. Within Bittacidae, males are readily distinguished from females, as the former display a modified terminalia, bearing a pair of epiandric appendages, an aedeagus with varying degrees of lengthening (called penisfilum when elongated, either coiled or uncoiled), a pair of reduced gonostyli (dististyles), a pair of pheromone producing eversible sacs between tergites VI and VII, and VII and VIII, as well as a characteristic flight pattern (Thornhill 1984, Byers 1996, Gao and Hua 2013). Yet, typically general appearance of both sexes is very similar. For this reason, an apparent case of sexual dimorphism appears worth describing. Moreover, a striking wasp-like appearance of the adult habitus, most remarkable in the male, also is worth noting.

## Methods

During examination of specimens at the National Collection of Insects of the Institute of Biology (CNIN-UNAM), a series of six specimens of *Eremobittacus spinulatus* immediately stood out because of the noticeable enlargement of the hind femora of males (less noticeable in females). Half of the specimens were female, allowing the first description of a female of this genus. Also, additional distribution data are presented for the species and observations on sexual dimorphism are discussed. Male and female specimens were dissected with a previous rehydration and terminalia were studied after clearing in 10% KOH at room temperature. Measurements were taken with a digital caliper and ranges are presented in mm with average in parentheses. Photographs were taken with an automontage system in a Zeiss Axio Zoom V16 stereomicroscope, while observations were made on a Zeiss Discovery V8 stereomicroscope.

## Results

### Eremobittacus spinulatus

Taxon classificationAnimaliaMecopteraBittacidae

Byers

Figs 1–6

#### Description.

**Figures 1–6.**
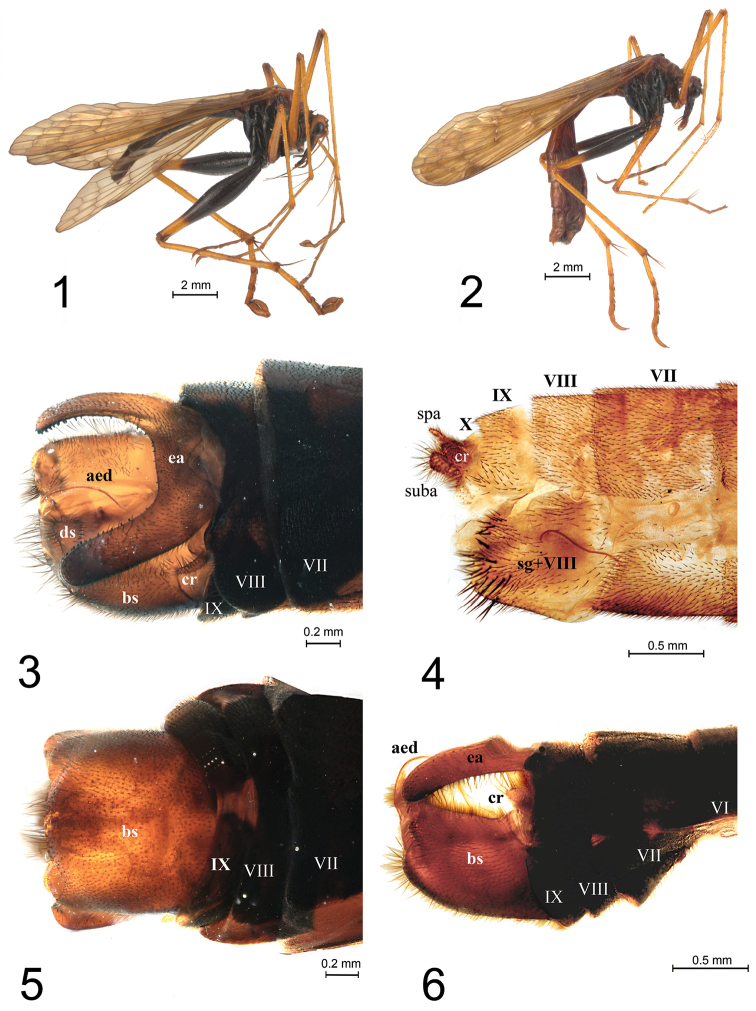
*Eremobittacus spinulatus* Byers. **1** Male habitus, lateral view **2** Female habitus, lateral view **3** Male genitalia, dorsal view **4** Female genitalia, lateral view **5** Male genitalia, ventral view **6** Same, lateral view. Abbreviations: aed, aedeagus; bs, basistyle; cr, cerci; ds, dististyle; ea, epiandrial appendage; spa, supra-anal plate (= XI tergum); suba, sub-anal plate (= XI sternum); sg, subgenital plate; roman number denotes abdominal segments.

*Male* (Fig. 1; *n* = 3, pinned). Forewing length 13.0–13.3 (13.2); forewing width 2.7–3.2 (3.0); antenna length 5.9-6.6 (6.2); hind femur length 6.0-6.4 (6.2); hind femur width 1.2–1.3 (1.25). Original description in Byers (1997).

*Female* (Fig. 2; *n* = 3, pinned). Forewing length 12.2–13.1(12.7); forewing width 3.0; antennae length 6.0–6.5 (6.2); hind femur length 5.1-5.4 (5.2); hind femur width 0.63–0.68 (0.65). General appearance similar to male, particularly in coloration and general body proportions, however hind femora appear narrower than in male (Fig. 1).

*Abdomen* (Fig. 4). Terga and pleura dark brown to entirely black, with dark short setae; VIII sternite fused with the subgenital plate, dorsally separated from tergites VIII and IX by an incision; stigma of segment VIII above a concavity in the sternite; subgenital plate with about 30 conspicuous dark spine-like setae, with an oblique plate connected with the 9th tergite; short non segmented cerci reaching about half the length of supra anal plate (or XI tergite).

*Legs* (Fig. 2). First and second pair of legs pale brown, with darkened areas at joints; hind legs modified, basal three fourths of femur blackish brown, distal fourth pale brown, slightly widened at median; widening about half that in males (Fig. 1), conveying sexual dimorphism; also, femur spine-like setae less developed than in males.

*Intraspecific variation*. In the original description by Byers (1997, fig. 11), the male epiandric appendix is illustrated with a slight median protuberance, dorsally; however, the protuberance is inconspicuous in the specimens examined (Fig. 3). Furthermore, a ventral view of the basistilum (*bs*) was not included in the original description (herein shown in Fig. 5) and its setation was illustrated only partially (herein shown in Figs 3, 5 and 6). One of the main diagnostic traits proposed for this species is a pattern of three transverse veins surrounded by a darkened region in both fore and hindwing; conversely, this feature was found variable, as in some specimens the pattern is diffuse or dimly visible. The wing longitudinal veins also display a certain degree of variation, for example, in number of veins between *R_2_* and *R_3_*, *R_3_* and *R_4_*, and between *R_5_* and *M_1_*, as well as in number of *Pcv* veins under the pterostigma.

#### Material examined.

Mexico, Oaxaca, 26 km SE Cuicatlán, 17°37'02.09"N, 96°55'23.52"W, 1080 m, 16-X-1998, M.A. Morales, 1♀; same data except E. Ramirez collector, 1♀; Oaxaca, 25 km SE Cuicatlán, 17°37'16.38"N, 96°55'10.02"W, 1000 m, 17-X-1998, F. Noguera, 1♀; Oaxaca, 26 km SSE Cuicatlán, 17°36'9.88"N, 96°55'39.2"W, 1080 m, 16-X-1998, E. Ramírez, 1♂; same data except M.A. Morales, 1♂; same data except 18-X1998, 1♂.

### Key to the species of *Eremobittacus* Byers

**Table:** 

1	Cuticle of whole body and hind femora with spiny surface, contrasting coloration present (black and orange), crossveins between *R* and *M* aligned transversely, epiandrial appendage subequal in thickness; Oaxaca and Puebla (Mexico)	***Eremobittacus spinulatus* Byers, 1997**
–	Cuticle of whole body and hind femora without spiny surface, contrasting coloration absent, crossveins between *R* and *M* not aligned transversely, epiandrial appendage noticeably reducing in thickness apically; Sinaloa (Mexico)	***Eremobittacus sodalium* Byers, 2011**

**Figure 7.**
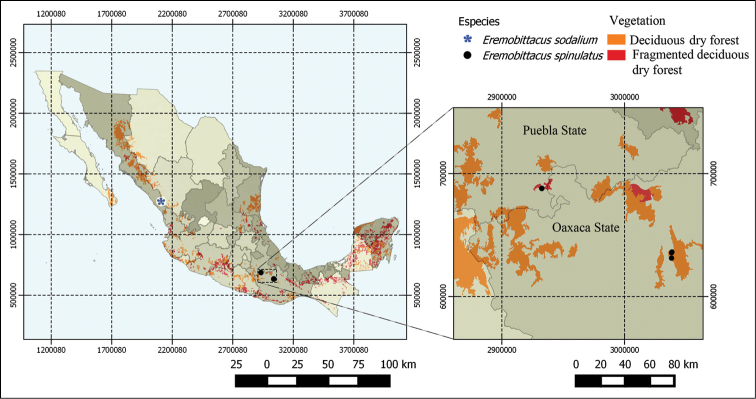
Present distribution of the genus *Eremobittacus* in Mexico.

Notes on distribution (Fig. 7). *Eremobittacus spinulatus* was known only from Puebla state (near Petlalcingo), east central Mexico. Herein, records for the state of Oaxaca (southeastern Mexico) are presented for the first time, increasing its distribution range in about 125 km. Both localities are within tropical dry forest, as is the type locality of *Eremobittacus sodalium* in the state of Sinaloa. This ecosystem in Mexico is characterized by its high biological diversity and also by a high degree of endemicity (Dirzo and Ceballos 2010, Noguera et al. 2012).

## Discussion

*Eremobittacus* (exemplified here with *Eremobittacus spinulatus*) appears to depart from a typical hangingfly habitus (i.e., similar to a cranefly, with long slender legs of subequal shape; Figs 1–2), the generalized condition in Bittacidae. From here, we believe two points should be made. First, there is a distinct sexual dimorphism, evidenced by thicker femora of hind legs in males, and to a less degree, a denser spiniform setation in male hind femora, as compared to females. Sexual dimorphism, to our knowledge, had not been recorded before for a bittacid. In itself, this phenomenon requires further study (e.g., morphometrics, behavior) for a more accurate description, as well as a possible explanation (e.g., sexual selection). According to Byers and Thornhill (1983), visual signals (e.g., wing and body movements) are important in close-range sexual interactions in *Panorpa* and bittacids, so a visual interaction between sexes is not disparate as a first working hypothesis that may explain a selective force leading to dimorphism in these species (e.g., female choice of males with thicker femora). This could be part of the customary nuptial gift of a prey item offered by males to females, in which females discriminate against males with unpalatable or small prey by flying away (Byers and Thornhill 1983).

Second, we suggest that the genus habitus is wasp-like (Figs 1, 2), similar to a sphecid (e.g., *Chalybion*, *Sceliphron*) or crabronid wasp. Again, this working hypothesis would require observation in nature in order to test whether behavior of the species agrees with such wasp-like resemblance (i.e., a case of mimicry). If in nature, *Eremobittacus* does not only behave as a typical hangingfly (e.g., a hanging food capturing strategy), but spends time capturing food in movement, as many bittacids do (Byers and Thornhill 1983), a wasplike, mimicry hypothesis would be supported. Although web-building spiders are frequent predators of Panorpidae and Bittacidae, they are also eaten by damselflies, robber flies, assassin bugs, and roving spiders (Byers and Thornhill 1983), some of which most likely would be discouraged by a wasp-looking potential prey (e.g., wasps have strong mandibles and sting). Recently, a case of mimicry has been made in fossil mecopterans (Wang et al. 2012). They mention a leaf mimesis of the hangingfly *Juracimbrophlebia ginkgofolia* Wang et al. (Cimbrophlebiidae) on a member of the Ginkgoales or ginkgos, *Yimaia capituliformis* (Yimaiaceae), from the Middle Jurassic of Mongolia. The insect wings resembled leaves in order to avoid predators (crypsis), or perhaps the insect provided an antiherbivore function for its plant hosts (mutualism). In *Eremobittacus*, a body robust and compact, with a slender abdomen attached to the thorax, a hind femur longer (and thicker) than other legs, and a potential warning coloration of the body, black and orange (as occurs in some wasps), may for the meantime represent a working hypothesis for another case of mimicry in the Mecoptera.

## Supplementary Material

XML Treatment for Eremobittacus spinulatus
